# 1-(2-Nitro­benz­yl)-1*H*-pyrrole-2-carbaldehyde

**DOI:** 10.1107/S1600536811018459

**Published:** 2011-05-20

**Authors:** Xu Chen, Ying Liu, Deng-Ke Liu, Ping-Bao Wang

**Affiliations:** a Henan University, Henan 475004, People’s Republic of China; b Tianjin Institute of Pharmaceutical Research, Tianjin 300193, People’s Republic of China

## Abstract

In the title compound, C_12_H_10_N_2_O_3_, the five- and six-membered rings form a dihedral angle of 83.96 (6)°. The nitro group is twisted by 5.92 (8)° from the plane of the attached benzene ring. In the crystal, weak inter­molecular C—H⋯O hydrogen bonds link the mol­ecules into columns in the [100] direction, with a short distance of 3.725 (3) Å between the centroids of benzene rings inside these columns.

## Related literature

The title compound is an inter­mediate in the synthesis of lixivaptan (systematic name *N*-[3-chloro-4-(5*H*-pyrrolo-[2,1-*c*][1,4]benzodiazepin-10(11*H*)-ylcarbon­yl)phen­yl]-5-fluoro-2-methyl­benzamide), a vasopressin receptor antagonist with high V2 receptor affinity. For preliminary studies of lixivaptan, which is now undergoing Phase III clinical trials, see, for example, Ku *et al.* (2009 ▶). For the synthesis of the title compound, see: Albright *et al.* (1998 ▶).
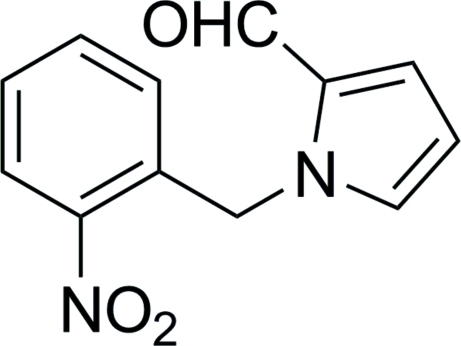


## Experimental

### 

#### Crystal data

C_12_H_10_N_2_O_3_
*M* *_r_* = 230.22
Triclinic, 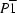

*a* = 7.2643 (8) Å
*b* = 8.3072 (10) Å
*c* = 9.2570 (12) Å
α = 104.10 (2)°
β = 96.463 (11)°
γ = 96.92 (2)°
*V* = 531.98 (11) Å^3^
*Z* = 2
Mo *K*α radiation
μ = 0.11 mm^−1^
*T* = 113 K
0.20 × 0.18 × 0.10 mm

#### Data collection

Rigaku Saturn CCD area-detector diffractometer
Absorption correction: multi-scan (*CrystalClear*; Rigaku/MSC, 2005 ▶) *T*_min_ = 0.979, *T*_max_ = 0.990
6867 measured reflections
2544 independent reflections
1517 reflections with *I* > 2σ(*I*)
*R*_int_ = 0.040

#### Refinement

*R*[*F* ^2^ > 2σ(*F* ^2^)] = 0.037
*wR*(*F* ^2^) = 0.095
*S* = 0.95
2544 reflections
154 parameters
H-atom parameters constrained
Δρ_max_ = 0.24 e Å^−3^
Δρ_min_ = −0.28 e Å^−3^

### 

Data collection: *CrystalClear* (Rigaku/MSC, 2005 ▶); cell refinement: *CrystalClear*; data reduction: *CrystalClear*; program(s) used to solve structure: *SHELXS97* (Sheldrick, 2008 ▶); program(s) used to refine structure: *SHELXL97* (Sheldrick, 2008 ▶); molecular graphics: *SHELXTL* (Sheldrick, 2008 ▶); software used to prepare material for publication: *SHELXTL*.

## Supplementary Material

Crystal structure: contains datablocks global, I. DOI: 10.1107/S1600536811018459/cv5092sup1.cif

Structure factors: contains datablocks I. DOI: 10.1107/S1600536811018459/cv5092Isup2.hkl

Supplementary material file. DOI: 10.1107/S1600536811018459/cv5092Isup3.cdx

Supplementary material file. DOI: 10.1107/S1600536811018459/cv5092Isup4.cml

Additional supplementary materials:  crystallographic information; 3D view; checkCIF report

## Figures and Tables

**Table 1:** Hydrogen-bond geometry (Å, °)

*D*—H⋯*A*	*D*—H	H⋯*A*	*D*⋯*A*	*D*—H⋯*A*
C2—H2⋯O3^i^	0.95	2.41	3.1919 (17)	139
C8—H8⋯O3^ii^	0.95	2.37	3.3107 (17)	170

Symmetry codes: (i) 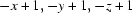; (ii) 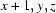.

**Fig. 1.**
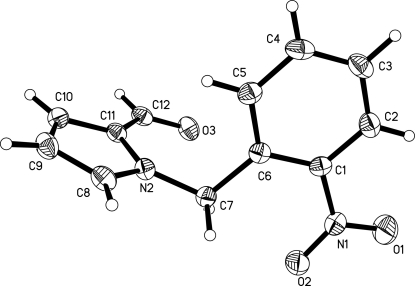
The molecular structure of (I), with the atomic numbering and 50% probability displacement ellipsoids.

## supplementary crystallographic information

### Comment

Lixivaptan is a vasopressin receptor antagonist with high V2 receptor affinity and is now undergoing Phase III clinical trials. Studies so far have demonstrated that Lixivaptan is efficacious in the correction of hyponatremia in SIADH, heart failure and liver cirrhosis with ascites, and few adverse effects have been noted (Ku *et al.*, 2009). Herewith we present the crystal structure of the title compound (I),  is an intermediate used in the synthesis of Lixivaptan.

In (I), the pyrrole ring and the benzene ring form a dihedral angle of 83.96 (6)°. Nitro group is twisted from the plane of attached benzene ring with a dihedral angle of 5.92 (8)°. In the crystal structure, weak intermolecular C—H···O hydrogen bonds (Table 1) further assemble these molecules into columns propagated in direction [100], with the short distance of 3.725 (3)Å between the centroids of benzene rings inside these columns.

### Experimental

(I) was prepared from pyrrole-2-carboxaldehyde and 2-nitrobenzyl bromide in the solution of sodium hydride in *N*,*N*-dimethylformamide under argon. Colourless crystals (m.p. 138 °C) were obtained in a yield of 95% as crude product. Single crystals were grown from petroleum ether-ethyl acetate (2:1) solution.

### Refinement

All H atoms were positioned geometrically and refined using a riding model, with d(C—H) = 0.95 - 0.99 Å and *U*_iso_(H) = 1.2*U*_eq_(C).

### Crystal data

**Table:**

C_12_H_10_N_2_O_3_	*Z* = 2
*M**_r_* = 230.22	*F*(000) = 240
Triclinic, *P*1	*D*_x_ = 1.437 Mg m^−^^3^
Hall symbol: -P 1	Mo *K*α radiation, λ = 0.71073 Å
*a* = 7.2643 (8) Å	Cell parameters from 1889 reflections
*b* = 8.3072 (10) Å	θ = 2.3–28.0°
*c* = 9.2570 (12) Å	µ = 0.11 mm^−^^1^
α = 104.10 (2)°	*T* = 113 K
β = 96.463 (11)°	Prism, colourless
γ = 96.92 (2)°	0.20 × 0.18 × 0.10 mm
*V* = 531.98 (11) Å^3^	

### Data collection

**Table:**

Rigaku Saturn CCD area-detector diffractometer	2544 independent reflections
Radiation source: rotating anode	1517 reflections with *I* > 2σ(*I*)
multilayer	*R*_int_ = 0.040
Detector resolution: 14.22 pixels mm^-1^	θ_max_ = 28.0°, θ_min_ = 2.3°
ω and φ scans	*h* = −9→9
Absorption correction: multi-scan (*CrystalClear*; Rigaku/MSC, 2005)	*k* = −10→10
*T*_min_ = 0.979, *T*_max_ = 0.990	*l* = −12→12
6867 measured reflections	

### Refinement

**Table:**

Refinement on *F*^2^	Primary atom site location: structure-invariant direct methods
Least-squares matrix: full	Secondary atom site location: difference Fourier map
*R*[*F*^2^ > 2σ(*F*^2^)] = 0.037	Hydrogen site location: inferred from neighbouring sites
*wR*(*F*^2^) = 0.095	H-atom parameters constrained
*S* = 0.95	*w* = 1/[σ^2^(*F*_o_^2^) + (0.0455*P*)^2^] where *P* = (*F*_o_^2^ + 2*F*_c_^2^)/3
2544 reflections	(Δ/σ)_max_ < 0.001
154 parameters	Δρ_max_ = 0.24 e Å^−^^3^
0 restraints	Δρ_min_ = −0.28 e Å^−^^3^

### Special details

**Table:**

Geometry. All e.s.d.'s (except the e.s.d. in the dihedral angle between two l.s. planes) are estimated using the full covariance matrix. The cell e.s.d.'s are taken into account individually in the estimation of e.s.d.'s in distances, angles and torsion angles; correlations between e.s.d.'s in cell parameters are only used when they are defined by crystal symmetry. An approximate (isotropic) treatment of cell e.s.d.'s is used for estimating e.s.d.'s involving l.s. planes.
Refinement. Refinement of *F*^2^ against ALL reflections. The weighted *R*-factor *wR* and goodness of fit *S* are based on *F*^2^, conventional *R*-factors *R* are based on *F*, with *F* set to zero for negative *F*^2^. The threshold expression of *F*^2^ > σ(*F*^2^) is used only for calculating *R*-factors(gt) *etc*. and is not relevant to the choice of reflections for refinement. *R*-factors based on *F*^2^ are statistically about twice as large as those based on *F*, and *R*- factors based on ALL data will be even larger.

### Fractional atomic coordinates and isotropic or equivalent isotropic displacement parameters (Å^2^)

**Table:**

	*x*	*y*	*z*	*U*_iso_*/*U*_eq_	
O1	0.73782 (17)	0.83214 (12)	0.55982 (12)	0.0507 (4)	
O2	0.80539 (16)	0.77297 (12)	0.33569 (12)	0.0402 (3)	
O3	0.39124 (13)	0.35491 (12)	0.08896 (11)	0.0281 (2)	
N1	0.76728 (15)	0.73149 (14)	0.44833 (13)	0.0255 (3)	
N2	0.78470 (15)	0.30999 (13)	0.04974 (12)	0.0206 (3)	
C1	0.75954 (17)	0.55405 (15)	0.45094 (15)	0.0202 (3)	
C2	0.73377 (18)	0.52045 (17)	0.58762 (15)	0.0258 (3)	
H2	0.7209	0.6085	0.6714	0.031*	
C3	0.72705 (19)	0.35811 (18)	0.60071 (16)	0.0291 (3)	
H3	0.7088	0.3334	0.6935	0.035*	
C4	0.74712 (19)	0.23132 (17)	0.47785 (16)	0.0284 (3)	
H4	0.7449	0.1196	0.4869	0.034*	
C5	0.77038 (18)	0.26669 (16)	0.34158 (16)	0.0244 (3)	
H5	0.7827	0.1777	0.2583	0.029*	
C6	0.77633 (17)	0.42904 (16)	0.32299 (14)	0.0198 (3)	
C7	0.79970 (19)	0.46275 (15)	0.17131 (14)	0.0220 (3)	
H7A	0.7028	0.5293	0.1451	0.026*	
H7B	0.9238	0.5311	0.1800	0.026*	
C8	0.93194 (19)	0.24383 (17)	−0.00619 (15)	0.0251 (3)	
H8	1.0605	0.2868	0.0300	0.030*	
C9	0.86490 (19)	0.10454 (17)	−0.12366 (15)	0.0275 (3)	
H9	0.9382	0.0351	−0.1829	0.033*	
C10	0.66954 (19)	0.08388 (15)	−0.14002 (15)	0.0237 (3)	
H10	0.5857	−0.0025	−0.2124	0.028*	
C11	0.61976 (18)	0.21166 (15)	−0.03203 (14)	0.0200 (3)	
C12	0.43411 (19)	0.24082 (17)	−0.00666 (15)	0.0226 (3)	
H12	0.3335	0.1635	−0.0708	0.027*	

### Atomic displacement parameters (Å^2^)

**Table:**

	*U*^11^	*U*^22^	*U*^33^	*U*^12^	*U*^13^	*U*^23^
O1	0.0839 (10)	0.0285 (6)	0.0404 (7)	0.0147 (6)	0.0260 (7)	−0.0002 (5)
O2	0.0676 (8)	0.0260 (6)	0.0292 (6)	0.0057 (5)	0.0086 (6)	0.0110 (5)
O3	0.0241 (5)	0.0363 (6)	0.0267 (6)	0.0097 (4)	0.0074 (4)	0.0094 (5)
N1	0.0252 (6)	0.0239 (6)	0.0257 (7)	0.0043 (5)	0.0009 (5)	0.0042 (5)
N2	0.0180 (6)	0.0239 (6)	0.0197 (6)	0.0037 (5)	0.0028 (5)	0.0054 (5)
C1	0.0153 (6)	0.0201 (7)	0.0250 (8)	0.0017 (5)	0.0001 (5)	0.0072 (6)
C2	0.0202 (7)	0.0340 (8)	0.0216 (8)	0.0021 (6)	0.0024 (6)	0.0056 (6)
C3	0.0245 (8)	0.0399 (9)	0.0249 (8)	0.0001 (7)	0.0004 (6)	0.0160 (7)
C4	0.0248 (8)	0.0274 (7)	0.0355 (9)	−0.0001 (6)	−0.0011 (6)	0.0172 (7)
C5	0.0226 (7)	0.0222 (7)	0.0273 (8)	0.0024 (6)	0.0001 (6)	0.0068 (6)
C6	0.0139 (6)	0.0229 (7)	0.0222 (7)	0.0020 (5)	0.0002 (5)	0.0065 (6)
C7	0.0236 (7)	0.0212 (7)	0.0203 (7)	0.0016 (6)	0.0019 (6)	0.0051 (6)
C8	0.0178 (7)	0.0332 (8)	0.0273 (8)	0.0065 (6)	0.0060 (6)	0.0114 (6)
C9	0.0295 (8)	0.0315 (8)	0.0260 (8)	0.0123 (6)	0.0098 (6)	0.0095 (6)
C10	0.0270 (7)	0.0225 (7)	0.0210 (7)	0.0017 (6)	0.0028 (6)	0.0058 (6)
C11	0.0192 (7)	0.0234 (7)	0.0183 (7)	0.0023 (5)	0.0020 (5)	0.0082 (5)
C12	0.0205 (7)	0.0278 (7)	0.0215 (7)	0.0024 (6)	0.0014 (6)	0.0116 (6)

### Geometric parameters (Å, °)

**Table:**

O1—N1	1.2175 (14)	C4—H4	0.9500
O2—N1	1.2247 (14)	C5—C6	1.3968 (18)
O3—C12	1.2260 (15)	C5—H5	0.9500
N1—C1	1.4745 (16)	C6—C7	1.5207 (17)
N2—C8	1.3571 (16)	C7—H7A	0.9900
N2—C11	1.3909 (16)	C7—H7B	0.9900
N2—C7	1.4602 (15)	C8—C9	1.3750 (19)
C1—C2	1.3881 (17)	C8—H8	0.9500
C1—C6	1.4000 (18)	C9—C10	1.3952 (18)
C2—C3	1.3787 (19)	C9—H9	0.9500
C2—H2	0.9500	C10—C11	1.3834 (17)
C3—C4	1.3837 (19)	C10—H10	0.9500
C3—H3	0.9500	C11—C12	1.4335 (17)
C4—C5	1.3867 (18)	C12—H12	0.9500
			
O1—N1—O2	122.35 (12)	C1—C6—C7	123.58 (11)
O1—N1—C1	118.45 (12)	N2—C7—C6	113.43 (10)
O2—N1—C1	119.20 (11)	N2—C7—H7A	108.9
C8—N2—C11	108.47 (11)	C6—C7—H7A	108.9
C8—N2—C7	125.06 (11)	N2—C7—H7B	108.9
C11—N2—C7	126.43 (10)	C6—C7—H7B	108.9
C2—C1—C6	122.84 (12)	H7A—C7—H7B	107.7
C2—C1—N1	115.52 (12)	N2—C8—C9	108.95 (12)
C6—C1—N1	121.64 (12)	N2—C8—H8	125.5
C3—C2—C1	119.42 (13)	C9—C8—H8	125.5
C3—C2—H2	120.3	C8—C9—C10	107.48 (12)
C1—C2—H2	120.3	C8—C9—H9	126.3
C2—C3—C4	119.60 (13)	C10—C9—H9	126.3
C2—C3—H3	120.2	C11—C10—C9	107.74 (12)
C4—C3—H3	120.2	C11—C10—H10	126.1
C3—C4—C5	120.23 (13)	C9—C10—H10	126.1
C3—C4—H4	119.9	C10—C11—N2	107.36 (11)
C5—C4—H4	119.9	C10—C11—C12	127.39 (13)
C4—C5—C6	122.05 (13)	N2—C11—C12	125.25 (12)
C4—C5—H5	119.0	O3—C12—C11	126.96 (13)
C6—C5—H5	119.0	O3—C12—H12	116.5
C5—C6—C1	115.84 (12)	C11—C12—H12	116.5
C5—C6—C7	120.58 (12)		
			
O1—N1—C1—C2	−5.10 (18)	C11—N2—C7—C6	82.11 (15)
O2—N1—C1—C2	173.91 (12)	C5—C6—C7—N2	9.06 (17)
O1—N1—C1—C6	174.59 (12)	C1—C6—C7—N2	−170.91 (11)
O2—N1—C1—C6	−6.41 (19)	C11—N2—C8—C9	0.35 (14)
C6—C1—C2—C3	1.07 (19)	C7—N2—C8—C9	−177.37 (12)
N1—C1—C2—C3	−179.24 (11)	N2—C8—C9—C10	−0.30 (15)
C1—C2—C3—C4	0.36 (19)	C8—C9—C10—C11	0.14 (15)
C2—C3—C4—C5	−1.2 (2)	C9—C10—C11—N2	0.08 (14)
C3—C4—C5—C6	0.6 (2)	C9—C10—C11—C12	179.91 (12)
C4—C5—C6—C1	0.72 (19)	C8—N2—C11—C10	−0.26 (14)
C4—C5—C6—C7	−179.26 (12)	C7—N2—C11—C10	177.42 (11)
C2—C1—C6—C5	−1.58 (19)	C8—N2—C11—C12	179.89 (12)
N1—C1—C6—C5	178.76 (11)	C7—N2—C11—C12	−2.4 (2)
C2—C1—C6—C7	178.40 (12)	C10—C11—C12—O3	−179.65 (12)
N1—C1—C6—C7	−1.26 (19)	N2—C11—C12—O3	0.2 (2)
C8—N2—C7—C6	−100.58 (14)		

### Hydrogen-bond geometry (Å, °)

**Table:**

*D*—H···*A*	*D*—H	H···*A*	*D*···*A*	*D*—H···*A*
C2—H2···O3^i^	0.95	2.41	3.1919 (17)	139
C8—H8···O3^ii^	0.95	2.37	3.3107 (17)	170

Symmetry codes: (i) −*x*+1, −*y*+1, −*z*+1; (ii) *x*+1, *y*, *z*.
